# Evaluation of the Novel mTA10 Selective Broth, MSB, for the Co-Enrichment and Detection of *Salmonella* spp., *Escherichia coli* O157 and *Listeria monocytogenes* in Ready-to-Eat Salad Samples

**DOI:** 10.3390/foods13010063

**Published:** 2023-12-23

**Authors:** Ana Costa-Ribeiro, Alexandre Lamas, Marta Prado, Alejandro Garrido-Maestu

**Affiliations:** 1 International Iberian Nanotechnology Laboratory, Av. Mestre José Veiga s/n, 4715-330 Braga, Portugal; ana.c.ribeiro@inl.int (A.C.-R.); marta.prado@inl.int (M.P.); 2 Department of Biochemistry, Genetics and Immunology, University of Vigo, 36310 Vigo, Spain; 3 Food Hygiene, Inspection and Control Laboratory (Lhica), Department of Analytical Chemistry, Nutrition and Bromatology, Veterinary School, Campus Terra, University of Santiago de Compostela (USC), 27002 Lugo, Spain; alexandre.lamas@usc.es

**Keywords:** selective enrichment, multiplex qPCR, *Salmonella* spp., *Escherichia coli* O157, *Listeria monocytogenes*

## Abstract

Multiplex assays implementing DNA-based methods have been demonstrated as suitable alternatives to culture-based microbiological methods; however, in most cases, they still require a suitable enrichment step. Finding suitable enrichment conditions for different bacteria may result in challenges. In the present study, a novel selective broth named MSB (mTA10 selective broth) was formulated for the simultaneous recovery of *Salmonella* spp., *E. coli* O157:H7 and *L. monocytogenes*. Attention was paid to ensure the optimal enrichment of *L. monocytogenes* as its enrichment is more challenging. To this end, cellobiose was added to increase the growth of *L. monocytogenes*, and sodium pyruvate was also added to improve the recovery of stressed bacteria. Four selective agents were added, namely nalidixic acid, sodium cholate, lithium chloride and potassium tellurite, to control the growth of interfering microorganisms. It was concluded that the novel broth was suitable for the simultaneous enrichment of the target pathogens, allowing them to reach concentrations higher than 7 log CFU/mL for each bacterium in pure culture. Furthermore, all heavily contaminated ready-to-eat salad samples reached concentrations higher than 5 log CFU/g. Finally, after 24 h of enrichment of spiked salad, it was possible to detect concentrations below 10 CFU/25 g.

## 1. Introduction

Infections associated with foodborne pathogens remain a major health issue in developed and developing countries. *Salmonella* spp., *E. coli* O157:H7 and *L. monocytogenes* are among the most relevant pathogens worldwide [1]. Official methods exist for the detection and control of these pathogens; however, these methods tend to be culture-based, and their limitations have been previously highlighted [2]. Likewise, the implementation of DNA-based methods such as real-time PCR (qPCR) has already been reported as a suitable alternative to the “classical” approach. Most qPCR-based methods still rely on an initial culture step to reach detectable levels of a given pathogen, as these tend to be in low concentrations in foods [3], which tends to allow us to reduce qPCR inhibitory components naturally present in foods [4] and also reduce the likelihood of false positive results due to the presence of DNA from dead bacteria [5]. This enrichment step is typically performed individually for each bacterium; however, this increases the cost per sample and the hands-on work. A reasonable alternative to overcome these issues would be to pursue multiplex methods, for which the challenge is to identify suitable culture conditions for bacteria with different physiological and metabolic characteristics such as those mentioned above [6]. Initial studies have addressed this problem via the development of non-selective media where these bacteria can be recovered, which is the case of the universal pre-enrichment broth (UPB) and simultaneous enrichment Broth (SEB) or the n17 broth later renamed as TA10 [7,8,9,10]. Additional studies reported media with improved performance by, for instance, reducing or removing the carbohydrates or changing the buffering components [11,12,13,14]. It is important to note that in many situations, these optimized formulations focus on the improved recovery of *L. monocytogenes*, which grows slower than the other two bacteria [9,15]. In addition to this, the use of selective media has also been explored, and media such as “*Salmonella*, *Escherichia*, and *Listeria*” (SEL), “*Salmonella enterica*, *Staphylococcus aureus*, and *Shigella dysenteriae*” (SSS) and/or “*Salmonella enterica*, *Staphylococcus aureus*, *Escherichia coli* O157: H7, and *Listeria monocytogenes*” (SSEL) among others have been reported to provide good results [15,16,17,18,19]. These are particularly useful when dealing with heavily contaminated samples [20]. This is the case for ready-to-eat (RTE) salad samples, which have become a common and convenient way of vegetable consumption and have been implicated in several cases of foodborne illnesses [21,22,23]. The constant search for better media for the recovery of these, and other, pathogens highlights the importance of this topic. The goal of the present study was to develop and evaluate a novel selective broth suitable for the simultaneous enrichment of *Salmonella* spp., *E. coli* O157:H7 and *L. monocytogenes* in RTE salad samples with a particular focus on *L. monocytogenes* because its characteristics are the most challenging for reliable detection in multiplex methods.

## 2. Materials and Methods

### 2.1. Bacterial Strains

*Salmonella enterica* serovar Typhimurium, WDCM 00031, *L. monocytogenes* WDCM 00021 and *E. coli* O157:H7 AMC 76 were selected as reference microorganisms for the evaluation of the novel selective broth and for spiking experiments. The first two were purchased from the Spanish Type Culture Collection, whereas the last was generously provided by the Institute of Applied Microbiology–ASMECRUZ. For all experiments, overnight fresh cultures were prepared by adding a single colony to Nutrient Broth (NB, Biokar Diagnostics S.A., Allonne, France), and the suspension was incubated at 37 °C. After incubation, the cultures were diluted and *Salmonella* spp. and *E. coli* were plated on Tryptic Soy Agar (TSA, Biokar Diagnostics S.A., Allonne, France), whereas *L. monocytogenes* was plated on Tryptic Soy Yeast Extract Agar (TSYEA, Biokar Diagnostics S.A., Allonne, France). All the plates were incubated overnight at 37 °C to determine the concentration of viable bacteria present in the spiking experiments.

ChromAgar™ Salmonella Plus, ChromAgar™ O157 (Chrom Salmonella and Chrom O157, respectively, CHROMagar Microbiology, Paris, France) and COMPASS Listeria (COMPASS, Biokar Diagnostics S.A., Allonne, France) were used as selective and differential media to determine the concentrations of *Salmonella* spp., *E. coli* O157 and *L. monocytogenes*, respectively, in mixed spiking experiments, as well as for result confirmation as detailed below. All the plates were incubated at 37 °C overnight and screened for typical colonies, mauve for *Salmonella* spp. and *E. coli* O157, and turquoise with halo for *L. monocytogenes* on the following day.

### 2.2. Selective Medium Formulation and Evaluation

**Figure 1:**
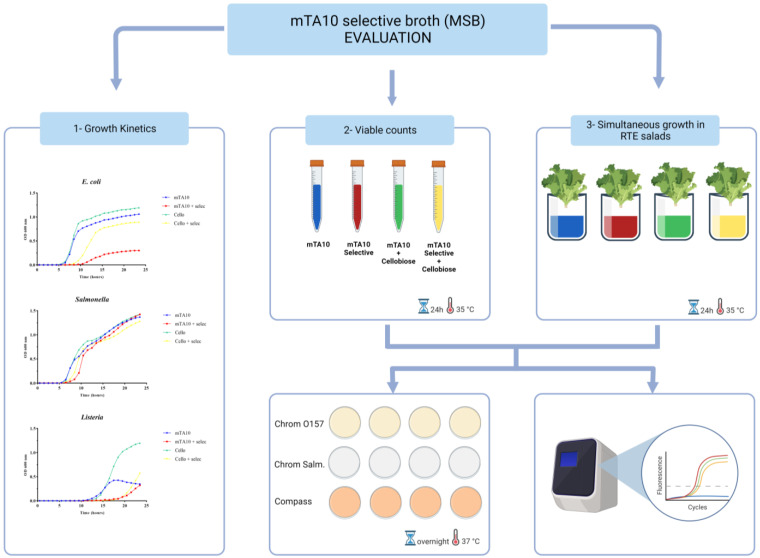
Schematic representation of the evaluation procedure of MSB.

The general broth mTA10 described by Garrido et al. [12] and later modified by Garrido-Maestu et al. [24] was selected as the basis for the novel selective broth. In order to enhance the recovery of stressed cells, *L. monocytogenes*, sodium pyruvate and cellobiose were added to the final formulation. Four selective agents were added to the basal medium, namely lithium chloride, nalidixic acid, potassium tellurite and sodium cholate. A panel of antimicrobials and their concentrations was selected based on previous studies to ensure the selection of optimal conditions [15,16,17,18,25,26]. This modified, selective formulation of mTA10 was renamed as MSB (mTA10 selective broth), and the final concentration of each component is detailed in Table 1. For comparison, the growth of the bacteria was also assessed in mTA10, mTA10 with cellobiose, and mTA10 with selective agents. In all experiments, the media were inoculated with 10–100 CFU of the corresponding microorganism and incubated at 35 °C for 24 h. From the mixed culture experiments, as described in Section 2.2.2 and Section 2.2.3, a 2 mL aliquot was taken for the DNA extraction (see Section 2.4), and multiplex qPCR analysis was conducted, as detailed below in Section 2.5. In Figure 1, a summarized workflow of the evaluation process is provided.

#### 2.2.1. Individual Bacterial Growth

The growth of different bacteria in different media was assessed by tracking the optical density at 600 nm (OD600), as previously described by Garrido-Maestu et al. [39,40]. Briefly, 200 µL of media were inoculated with the corresponding bacteria, prepared as detailed in Section 2.1, and the OD600 was measured every 30 min in a microplate reader for 24 h (Synergy H1, Biotek, Vinooski, VT, USA). From the data obtained, it was possible to calculate OD_max_, µ_max_ and λ, which represent the maximum optical density, the maximum specific growth rate and the lag time, respectively.

#### 2.2.2. Mixed Bacterial Cultures Growth

An individual, initial evaluation of the growth of each microorganism in a mixed culture was performed by spiking 10–100 CFU into 10 mL of each of the broths detailed above. After incubation, one hundred-fold serial dilutions were performed and plated on Chrom Salmonella, Chrom O157 and COMPASS. The selective agar media were incubated at 37 °C overnight, and the typical colonies were enumerated. 

#### 2.2.3. Evaluation of the Growth Capacity of Mixed Bacterial Cultures in Spiked RTE Salads

The same procedure described in Section 2.2.2 was followed, but instead of inoculating 10 mL of the different broths, 5 g of RTE salads were spiked and mixed with 45 mL of the corresponding media and homogenized for 30 s in a Stomacher 400 Circulator (Seward Limited, West Sussex, UK), after which the samples were incubated as detailed above and then serially diluted one hundred-fold to be plated on the different chromogenic media as previously described. This procedure was repeated in triplicate. 

### 2.3. RTE Salad Sample Inoculation and Processing in the New Method Implementing MSB

A sample size of 25 g was used, unless otherwise stated, as specified in the European Regulation 2073 [41]. These were spiked with freshly prepared bacterial cultures, as previously detailed in Section 2.1, and then 225 mL of MSB was added. The mixture was homogenized for 30 s in a Stomacher 400 Circulator (Seward Limited, West Sussex, UK) and incubated at 35 °C for 24 h. After enrichment, 2 mL was taken for DNA extraction as described in Section 2.4, and a loopful was streaked on the different chromogenic media.

### 2.4. DNA Extraction

The DNA extraction from spiked RTE samples and mixed cultures from Section 2.2.2 and Section 2.2.3 was performed by taking 2 mL aliquots, which were centrifuged at 900× *g* for 1 min only in case of spiked salads. The supernatant was recovered, placed in a new 2 mL tube and centrifuged at 16,000× *g* for 5 min. The pellet obtained was resuspended in 1 mL of TE buffer (TE 1X, Tris-HCl 10 mM, EDTA 1 mM) and then it was centrifuged again under the same conditions. The supernatant was discarded, and the resulting pellet was resuspended in 200 µL of lysozyme–achromopeptidase (20 mg/mL of lysozyme and 1 mg/mL of achromopeptidase prepared in TE 2X with 1.2% of Triton X-100 (Sigma–Aldrich, St. Louis, MO, USA)) and 25 µL of proteinase K. The suspension was incubated at 37 °C for 20 min. Upon completion, 400 µL of buffer CD1 from the DNeasy PowerSoil Pro kit (Qiagen, Barcelona, Spain) was added, incubated at 65 °C for 10 min and finally vortexed for 10 min. Subsequent steps were followed as described in the protocol provided by the manufacturer. The elution of the DNA was performed in 30 µL of elution buffer, which was passed twice through the filter by centrifuging at 15,000× *g* for 1 min, and the filtrate was recovered and re-centrifuged under the same conditions. The DNA extracts were stored at −20 °C until the analysis.

### 2.5. Pathogen Detection by Multiplex qPCR

The detection of each one of the different pathogens was performed using multiplex qPCR. The reactions were performed in a final volume of 20 µL with 10 µL of TaqMan^®^ Multiplex Master Mix (Applied Biosystems™, Foster City, CA, USA), 100 nM of *ttr* primers and probe, 200 and 150 nM of *hly* primers and probes, respectively, and 800 nM with a 400 nM probe targeting *rfbE*. In addition to these, 100 nM primers and probes were added for the detection of a non-competitive internal amplification control (IAC), along with 685 copies of IAC DNA (the sequences of all the primers and probes are provided in Table 2). A total of 3 µL of template DNA was loaded into every reaction, and the remaining volume was filled with nuclease-free water (New England BioLabs, Inc., Ipswich, MA, USA). The reactions were run in a QuantStudio™ 5 System (Applied Biosystems™, Foster City, CA, USA) with a thermal profile consisting of a hot-start step at 95 °C for 2 min, followed by 50 cycles of dissociation at 95 °C for 5 s and combined annealing–extension at 61 °C for 30 s. Samples with Cq values lower than 38 were considered positive and confirmed by plating in Chrom, as previously described. 

### 2.6. Fitness-for-Purpose

To serve as a proof of principle, and in order to better assess the performance of the method developed, a set of fifteen samples was spiked with decreasing concentrations of each of the pathogens at various concentrations. In addition to these, two non-spiked RTE salad samples were also included in the analysis to serve as negative controls. Once analyzed, each spiked sample reporting a positive result was classified as a Positive Agreement (PA), whereas if the result was negative, it was classified as a Negative Deviation (ND). In a similar way, non-spiked samples achieving a negative qPCR result were classified as Negative Agreement (NA); however, if the result was positive, it was classified as Positive Deviation (PD). The data obtained were used to calculate the relative sensitivity, specificity and accuracy using the formulae previously reported by Anderson et al. and Tomás et al. (SE, SP and AC, respectively) [46,47].

SE = PA/(PA + ND) × 100(1)

SP = NA/(NA + PD) × 100(2)

AC = [(PA + NA)/N] × 100(3)

N is the number of samples analyzed

### 2.7. Data Representation

Graphical representation of the data was performed using GraphPad Prism version 8.0.0 for Windows (GraphPad Software, San Diego, CA, USA, www.graphpad.com). 

## 3. Results

### 3.1. MSB Evaluation

#### 3.1.1. Individual Bacterial Growth

**Figure 2:**
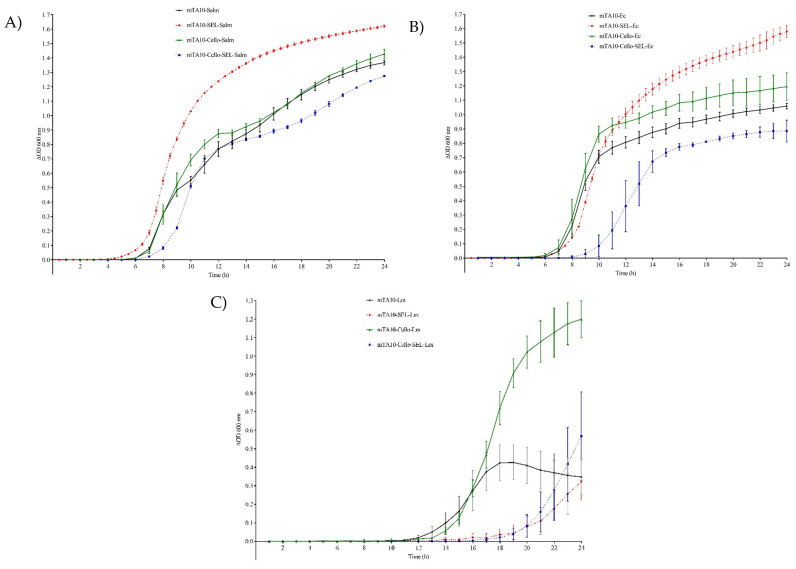
Individual growth kinetics of *Salmonella* spp. for 24 h at 35 °C (**A**), *E. coli* O157:H7 (**B**) and *L. monocytogenes* (**C**) in mTA10, mTA10 supplemented with cellobiose, mTA10 supplemented with selective agents and mTA10 with cellobiose and selective agents.

As shown in Figure 2A,B and Table 3, *Salmonella* spp. was the fastest-growing pathogen regardless of the broth formulation selected, followed by *E. coli* O157:H7, with *L. monocytogenes* being the slowest (Figure 2C). For both *Enterobacteriaceae*, the supplementation of broth with cellobiose did not cause any significant effect, whereas the addition of the selective agents only generated a minor extension of the lag phase, which was not relevant considering that the enrichment step lasted 24 h. In addition to this, the maximum absorbance values in all cases were ~1. More profound effects were observed in the growth of *L. monocytogenes* where the supplementation of the general formulation with cellobiose significantly increased the maximum OD600, with a minor effect on the lag phase, and the addition of the selective agents, just like with the other two pathogens, extended the lag phase, but the combined effect with cellobiose resulted in reaching a maximum OD600 of ~0.5, as shown in Figure 2C.

#### 3.1.2. Mixed Bacterial Cultures Growth 

**Figure 3:**
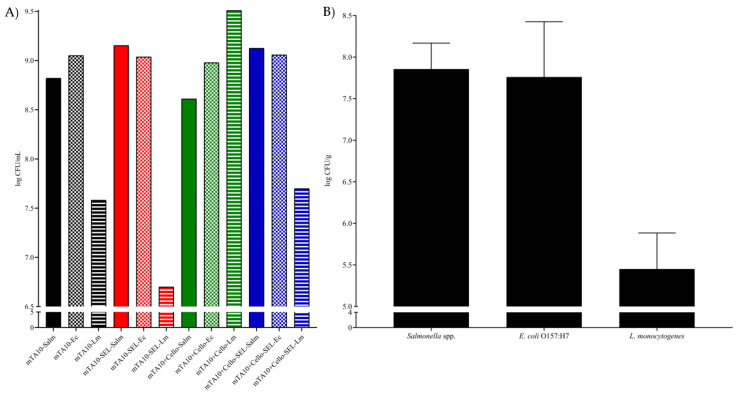
Bacterial concentration in log CFU/mL, or g, after 24 h of enrichment at 35 °C of mixed cultures of *Salmonella* spp., *E. coli* O157:H7 and *L. monocytogenes* in pristine MSB (**A**), and mixed cultures of *Salmonella* spp., *E. coli* O157:H7 and *L. monocytogenes* in spiked RTE salad samples (**B**).

All three target microorganisms were successfully enriched simultaneously in the different media combinations tested, as it was possible to reach final concentrations higher than 6 log CFU/mL in all cases. More specifically, *Salmonella* spp. reached values of 8.8, 9.1, 8.6 and 9. 1 log CFU/mL when enriched in mTA10, mTA10 with selective agents, mTA10 with cellobiose and MSB; thus, no major differences were observed. In regard to *E. coli* O157:H7, results similar to those of *Salmonella* spp. were observed, with final concentrations of 9.1, 9.0, 9.0 and 9.0 log CFU/mL for the corresponding media. More variability was observed in the enumeration of *L. monocytogenes*; as for mTA10, a concentration of 7.6 log CFU/mL was obtained, but this decreased to 6.7 log CFU/mL when the selective supplements were added; however, supplementing the medium with cellobiose resulted in a final concentration of 9.5 log CFU/mL, and once more, the addition of the selective agents decreased the final counts to 7.7 log CFU/mL. These data are summarized in Figure 3A.

#### 3.1.3. Mixed Bacterial Culture Growth in MSB Spiked RTE Salads

The three salad samples were spiked with 19, 10 and 46 CFU of *Salmonella* spp., *E. coli* O157:H7 and *L. monocytogenes*, respectively. After enrichment at 35 °C for 24 h, *Salmonella* spp. reached a concentration of 7.9 ± 0.3 log CFU/mL, *E. coli* O157:H7 7.8 ± 0.7 log CFU/mL and *L. monocytogenes* 5.5 ± 0.4 log CFU/mL. These data are summarized in Figure 3B.

### 3.2. Fitness-for-Purpose

The two negative samples included in the current study reported the expected negative result by multiplex qPCR, and no typical colonies were observed in any of the chromogenic media. In regard to the spiked samples, they were clustered into five different concentrations for each pathogen. In this regard, the inoculation levels obtained for *Salmonella* spp. ranged from 3.5 to 19 CFU/25 g; for *E. coli* O157:H7, it ranged from 3.8 to 9.6 CFU/25 g; and finally, for *L. monocytogenes*, the concentrations ranged from 3.7 to 46 CFU/25 g. For *Salmonella* spp., only one negative deviation was observed in a sample spiked with 3.5 CFU/25 g; however, no typical colonies were observed on the chromogenic agar. For *E. coli* O157:H7, two NDs were obtained, both in samples inoculated with 8.6 CFU/25 g, and similar to *Salmonella* spp., no typical colonies were observed on the chromogenic medium. Lastly, in regard to the analysis of *L. monocytogenes*, in line with the other pathogens, 1 ND was recorded in a sample spiked with 9.4 CFU/25 g. These data are presented in Table 4.

## 4. Discussion

Multiplex detection of pathogenic bacteria is a convenient way to increase laboratory throughput, thereby reducing hands-on work and the cost of analyses. Even though it has many advantages, this approach may be challenging because of the need to find suitable conditions for all the pathogens of interest. The first step for such a method to be developed is to identify a suitable enrichment broth for all the microorganisms of interest. *Salmonella* spp., *E. coli* O157:H7 and *L. monocytogenes* are among the most relevant bacterial pathogens worldwide [48,49]. Out of these, due to its characteristics, *L. monocytogenes* tends to be the most problematic to integrate with the mentioned *Enterobacteriaceae* using a common method, which is why mTA10, a general broth previously reported to provide good results for *L. monocytogenes*, was selected [14], and this was further improved by supplementing the broth with cellobiose, a carbohydrate only metabolized by *Listeria* and not by *Salmonella* or *E. coli*. This observation was confirmed via pure culture bacterial enrichment because the addition of this carbohydrate did not affect any of the growth parameters evaluated for *Salmonella* or *E. coli* (Figure 2A,B), but it significantly improved the final concentration of *L. monocytogenes* (Figure 2C). The capacity of the broth for the recovery of stressed bacteria was improved by supplementing it with sodium pyruvate (NaPyr) [34].

RTE salad samples are a popular way of vegetable intake and have also been identified as carriers of different pathogens [22,50,51]. This type of product has been reported to contain high concentrations of mesophilic bacteria [21,23]. For this reason, even though the improved formulation of the broth seemed to provide good results, the implementation of a selective formulation was expected to provide better results. After careful evaluation of previous studies [15,17,18,25,26,52] a set of four antimicrobials was selected, namely potassium tellurite, lithium chloride, nalidixic acid and sodium cholate. It was observed that the range of concentrations used in previous studies was relatively wide for each of the antimicrobials. Considering this situation, and to avoid having an excessively selective medium that may have some degree of inhibitory effect on the target bacteria, or a medium with low selectivity that leads to the growth of interfering bacteria, an intermediate concentration for each of them was selected. It is worth noting that instead of “bile salts” or “bile salts No 3”, sodium cholate was selected for the current formulation because this is a better-defined component, and was previously reported to not exert an inhibitory effect even in stressed *E. coli*. Thus, it was expected to perform better in the final formulation [37,53]. 

When analyzing the maximum OD600 obtained from each pure culture performed in Section 2.2.1, it was observed that the final values of the selective formulation were in the range of 0.5–1, and this value correlated to ~8 log CFU/mL [54]. This final concentration was very similar to the one obtained after mixed culture enrichment, as described in Section 2.2.2, which may be observed in Figure 3A, and the addition of the selective agents significantly impacted the growth of the three pathogens. However, it was still possible to reach final concentrations higher than 7 log CFU/mL when cultured together, similar to those reported for other selective multiplex broths [17,19,26,55]. A more profound effect was observed when the final bacterial concentration was determined in spiked RTE salad samples, as detailed in Section 2.2.3, where roughly a 2 log CFU/g reduction was observed, as shown in Figure 3B. These results are reasonable, considering the high concentrations of interfering bacteria expected in this type of food product, where up to 7 log CFU/g were previously reported [22,50,51]. Of the utmost importance is the fact that all three pathogens reached concentrations higher than 5 log CFU/g and returned positive results via multiplex qPCR.

Finally, when evaluating the performance of the method in a larger number of samples co-spiked with all three pathogens at different concentrations, the first thing to notice is that it was possible to detect all three pathogens at concentrations below 10 CFU/25 g; more specifically, the lowest concentrations detected were 3.5, 3.7 and 3.8 CFU/25 g for *Salmonella* spp., *E. coli* O157:H7 and *L. monocytogenes*, respectively. These concentrations are similar to those reported previously for other non-selective enrichment-based multiplex methods, such as those based on mTA10, TSB and/or SEB [8,12,56,57], or selective methods like SEL, SSEL or SSL [17,19,26,55]. Regarding the results obtained, only one ND was recorded for *Salmonella* spp. and *L. monocytogenes* and two for *E. coli* O157:H7. In terms of *Salmonella* spp., this deviation was most likely associated with a low inoculation level, 3.5 CFU/25 g. When focusing on the deviations identified for *L. monocytogenes* and *E. coli* O157:H7, in addition to being spiked with low concentrations below 10 CFU/25 g, it was observed that the Cq values obtained for the other two pathogens were very low; thus, there must be competence for the qPCR resources which may ultimately affect the amplification efficiency of the least represented pathogen, as already highlighted by Osman et al. and Compston et al. [58,59]. As an example, both NDs obtained for *E. coli* O157:H7 were inoculated with 8.6 CFU/25 g, and the Cq values obtained for *ttr* and *hly* were 19.7 and 25.6 in the first deviation, and 20.8 and 25.9 in the second. Similarly to what was described earlier, in the ND identified for *L. monocytogenes*, the specific sample was spiked with 9.4 CFU/25 g, and the Cq values of *ttr* and *rfbE* were 19.7 and 25.2, respectively. This was considered a minor limitation of the method as, from the food safety point of view, these deviations were all associated with very low initial bacterial concentrations as indicated above, which were all below 10 CFU/25 g, and additionally, two of the three pathogens of interest were successfully detected; thus, these products would have never reached the consumers.

## 5. Conclusions

A new selective enrichment broth, named MSB, suitable for the multiplex enrichment of *Salmonella* spp., *E. coli* O157:H7 and *L. monocytogenes*, was successfully formulated. The MSB obtained good results for the enrichment of all three pathogens in pure and mixed cultures as well as in spiked RTE salads with high concentrations of background microorganisms. Additional studies will be performed to further evaluate the performance of MSB in the recovery of these pathogens in other food matrices.

## Figures and Tables

**Table 1:** MSB composition.

Component	g/L	Function	Reference
Tryptose	10.0	Provides amino acids, especially essential amino acids, large peptides and other nitrogenous substances.	[27,28]
Beef extract	5.0	Provides peptides, amino acids, nucleotides, organic acids, minerals and vitamins.	
Yeast extract	5.0	Source of amino acids, peptides and water-soluble vitamins such as B12 complex and carbohydrates.	
NaCl	5.0	Maintain osmotic balance.	[29,30]
MOPS	8.5	Buffering agents.	[31,32]
MOPS-Na	13.7		
Cellobiose	5.0	Reducing sugar, which can be uptake by *Listeria monocytogenes* and used as a source of energy.	[33]
NaPyr	1.1	Used as an additional source of energy; bacterial growth inducer; free radical scavenger and reactive oxygen species (ROS)-quencher.	[34]
Lithium chloride	1.0	Broad-range inhibition of Gram-positive and Gram-negative bacteria.	[17,35,36]
Nalidixic acid	0.0025	Inhibition of competitive microbiota (able to inhibit microbiota that grow in the presence of LiCl).	[35]
Sodium cholate	1.0	Water-soluble bile salt that acts as a selective inhibitor. This substance interferes with the growth and incorporation of glucose and also inhibits flagellum formation in Gram-negative bacteria.	[37]
Potassium tellurite	0.0001	Inhibition of competitive microbiota due to its oxidative capacity (inhibits Gram-negative bacteria and some Gram-positive bacteria unable to use).	[38]

All the components, except for potassium tellurite, were dissolved in 1 L of milliQ water and sterilized via autoclaving. A stock of potassium tellurite was prepared and sterilized by filtering it through a 0.22 µm membrane; it was added to the final medium at room temperature. The final pH of the MSB was 7.2 ± 0.2.

**Table 2:** Multiplex qPCR primers and probes.

Microorganism	Primer	Sequence 5′ → 3′	Concentration (nM)	Modifications	Reference
*Salmonella* spp.	ttr-P3F	GGC TAA TTT AAC CCG TCG TCA G	100		[39]
	ttr-P3R	GTT TCG CCA CAT CAC GGT AGC	100		
	ttr-P3P	AAG TCG GTC TCG CCG TCG GTG	100	NED/MGBNFQ	
*L. monocytogenes*	hly-P3F	CGC AAC AAA CTG AAG CAA AGG A	200		[42,43]
	hly-P3R	CGA TTG GCG TCT TAG GAC TTG C	200		
	hly-P3P	CAT GGC ACC//ACC AGC ATC TCC G	150	FAM/ZEN/IABkFQ	
*E. coli* O157	O157-rfbE-F	TCA ACA GTC TTG TAC AAG TCC AC	800	-	[44]
	O157-rfbE-R	ACT GGC CTT GTT TCG ATG AG	800	-	
	O157-rfbE-P	AC TAG GAC CGC AGA GGA AAG AGA GGA A	400	Cy5/IAbRQSp	
-	NC-IAC-F	AGT TGC ACA CAG TTA GTT CGA G	100	-	[24]
	NC-IAC-R	TGG AGT GCT GGA CGA TTT GAA G	100	-	
	IAC-P	AGT GGC GGT//GAC ACT GTT GAC CT	100	YY/ZEN/IABkFQ	[45]

YY (Yakima Yellow), IABkFQ (Iowa Black^®^FQ), IAbRQSp (Iowa Black^®^Sp) and ZEN (secondary, internal quencher) are trademarks from IDT.

**Table 3:** Growth kinetics data.

	*Salmonella* spp.				*E. coli* O157:H7				*L. monocytogenes*			
	mTA10		mTA10 + Cello		mTA10		mTA10 + Cello		mTA10		mTA10 + Cello	
	N	S	N	S	N	S	N	S	N	S	N	S
OD_600_max	1.39 ± 0.02	1.55 ± 0.01	1.37 ± 0.06	1.17 ± 0.01	0.99 ± 0.02	1.48 ± 0.04	1.12 ± 0.09	0.87 ± 0.04	0.39 ± 0.10	0.32 ± 0.10	1.23 ± 0.13	0.57 ± 0.24
µmax	0.12 ± 0.01	0.25 ± 0.00	0.13 ± 0.01	0.13 ± 0.00	0.20 ± 0.02	0.21 ± 0.01	0.27 ± 0.05	0.18 ± 0.01	0.13 ± 0.01	0.07 ± 0.00	0.23 ± 0.02	0.16 ± 0.04
λ	5.71 ± 0.07	5.92 ± 0.09	5.58 ± 0.16	6.73 ± 0.02	6.67 ± 0.05	7.04 ± 0.03	6.79 ± 0.64	10.01 ± 0.90	13.56 ± 0.41	19.25 ± 1.34	14.76 ± 0.36	20.18 ± 0.90

N: normal, non-selective broth. S: broth with the four selective agents. “Cello” denotes medium supplemented with cellobiose.

**Table 4:** Sample spiking results.

Microorganism	Inoculation Level *	N	PA	NA	PD	ND	SE	SP	AC
*Salmonella* spp.	19	3	3				93	100	94
	10	3	3						
	5	5	5						
	3.5	4	3			1			
	0	2		2	0				
*E. coli* O157:H7	9.6	3	3				86	100	88
	8.6	5	3			2			
	6.4	4	4						
	3.8	3	3						
	0	2		2	0				
*L. monocytogenes*	46	3	3				93	100	94
	31	5	5						
	9.4	4	3			1			
	3.7	3	3						
	0	2		2	0				

* Values reported in CFU/25 g. N: Number of samples spiked at a given level. PA: Positive Agreement. NA: Negative Agreement. PD: Positive Deviation. ND: Negative Deviation. SE: Relative Sensitivity. SP: Relative Specificity. AC: Relative Accuracy. SE, SP and AC results expressed as %.

## Data Availability

The data used to support the findings of this study can be made available by the corresponding author upon request.
